# Rapid selection for resistance to diamide insecticides in *Plutella xylostella* via specific amino acid polymorphisms in the ryanodine receptor

**DOI:** 10.1016/j.neuro.2016.05.012

**Published:** 2017-05

**Authors:** Bartlomiej J. Troczka, Martin S. Williamson, Linda M. Field, T.G.Emyr Davies

**Affiliations:** Biological Chemistry and Crop Protection Department, Rothamsted Research, Harpenden, Hertfordshire, AL5 2JQ, UK

**Keywords:** Ryanodine receptor, Diamides, *Plutella xylostella*, Diamondback moth, Cruciferous crops

## Abstract

Diamide insecticides, such as flubendiamide and chlorantraniliprole, are a new class of insecticide with a novel mode of action, selectively activating the insect ryanodine receptor (RyR). They are particularly active against lepidopteran pests of cruciferous vegetable crops, including the diamondback moth, *Plutella xylostella*. However, within a relatively short period following their commercialisation, a comparatively large number of control failures have been reported in the field. In this review we summarise the current body of knowledge regarding the molecular mechanisms of diamide resistance in *P. xylostella*. Resistant phenotypes collected from different countries can often be linked to specific target-site mutation(s) in the ryanodine receptors’ transmembrane domain. Metabolic mechanisms of resistance have also been proposed. Rapid resistance development is probably a consequence of over-reliance on this one class of chemistry for diamondback moth control.

## Highlights

• Baseline susceptibility of *Plutella xylostella* to diamide insecticides collated.
• Instances of diamide resistance in *P. xylostella* summarized.
• Diamide insecticide specificity for the insect ryanodine receptor highlighted.
• Efforts to isolate and characterize the *P. xylostella* ryanodine receptor described.
• Molecular mechanisms of diamide resistance in *P. xylostella* discussed.

## Graphical abstract

**Figure:**
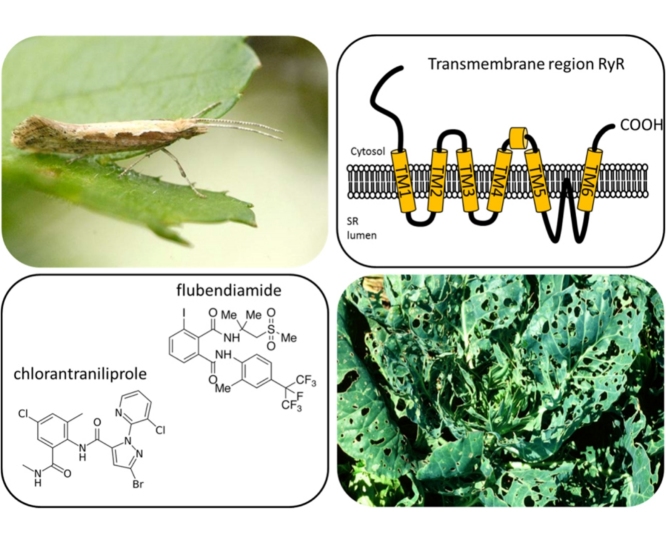

## 1 Background

The diamondback moth, *Plutella xylostella* (L.), is one of the most destructive insect pests of cruciferous vegetables, currently accounting for US$2.7 billion worth of annual worldwide crop losses (Zalucki et al., 2012). Most damage is caused by the caterpillars tunneling into the heads and/or foliage of plants such as cabbage, kale, swede, turnip and brussels sprouts. In addition, they can contaminate produce by pupating inside broccoli florets and cauliflower curds. Seedlings of cruciferous forage crops and oilseed rape may well be destroyed by this pest and severe defoliation or pod grazing can also significantly reduce oilseed rape yield. Control costs associated with this pest are in the region of US$1.3 billion to US$2.3 billion annually (Zalucki et al., 2012). Typically, control of this pest depends solely on the use of synthetic insecticides.

Flubendiamide is an extremely effective insecticide against *P. xylostella*, especially when used as a larvicide (Tohnishi et al., 2005, Nauen, 2006, Hirooka et al., 2007). The parent compound structure was discovered by Nihon Nohyaku Co., Ltd during their pyrazinedicarboxamide herbicide development program conducted in the early 1990s. The discovery of more potent substituents led to the synthesis, in 1998, of a phthalic acid diamide insecticide, later named flubendiamide (Nishimatsu et al., 2005), co-developed by Nihon Nohyaku and Bayer CropScience AG (Nauen, 2006, Tsubata et al., 2007). Flubendiamide has an excellent biological and ecological profile (Hilder and Boulter, 1999, Hall, 2007) and a favourable toxicological profile (Ebbinghaus-Kintscher et al., 2006). The first registration was secured in the Philippines in 2006 and was followed a year later by successful registrations in Japan, Pakistan, Chile, India and Thailand under the trade names Amoli^®^, Belt^®^, Fame^®^, Fenos^®^, Synapse^®^, Phoenix^®^ and Takumi^^®^^ (Hirooka et al., 2007). Flubendiamide was classified as the first member of the new group 28 (ryanodine receptor modulator) insecticides within the IRAC (Insecticide Resistance Action Committee) mode of action classification scheme (Nauen, 2006). This scheme, developed to provide guidance on resistance management strategies, facilitates the alternation of compounds belonging to different groups in order to delay or avoid the rapid development of resistance in treated pest insects.

Chlorantraniliprole or Rynaxypyr^®^ (Dupont, USA), is another insecticide in the IRAC Mode of Action Group 28 family. Chlorantraniliprole was the first member of the anthranilic diamides, and, as with flubendiamide, is particularly effective for control of insects in the order Lepidoptera (Temple et al., 2009). Chlorantraniliprole is relatively harmless to beneficial arthropods and was not found to exhibit cross resistance with existing insecticides (Lahm et al., 2009). Products containing this active ingredient were launched on the world market in 2007. This insecticide is currently sold under the trade names Acelepryn^®^, Altacor^®^, Coragen^®^, Dermacor^®^ X-100, Prevathon^®^, Voliam^®^ Flexi and Voliam^®^ Xpress Durivo^®^ and Virtako^®^. Cyantraniliprole or Cyazypyr™, a second anthranilic diamide discovered by DuPont and co-developed with Syngenta (Wiles et al., 2011), is chemically similar to chlorantraniliprole, but exhibits a broader spectrum insecticidal activity and provides good control of sucking and piercing insects such as aphids and whiteflies (Foster et al., 2012, Gravalos et al., 2015). The broad spectrum of this anthranilic diamide is thought to be due to its physical properties, i.e a lower log*P* and higher water solubility, in comparison to the other diamide insecticides, making it more suitable for systemic applications (Selby et al., 2013). Products containing cyantraniliprole were launched in selected countries from 2012 under the trade names Exirel^®^, Verimark^®^, Ference^®^, Fortenza Duo^®^, Benevia^®^ and Spinner^®^.

## 2 Baseline susceptibility monitoring for diamide insecticides

The LC_50_ value (lethal concentration that provides 50% mortality) of a particular insecticide can be used to establish a baseline susceptibility for a target population. This can then be used as a baseline reference in future monitoring surveys to determine if the susceptibility of the target population has shifted after the population has been exposed to the insecticide. Actual LC_50_ values can be compared between populations by examining the 95% confidence intervals, whereby if the upper and lower limits do not overlap then it is likely that the population has experienced a significant loss of susceptibility. Such a change could be indicative of a resistance problem and should trigger further investigation. Baseline monitoring for *P. xylostella* susceptibility to chlorantraniliprole was conducted by DuPont in the Philippines from 2006 to 2008, at locations in Benguet Province (Buguias and La Trinidad) and Laguna Province (Calauan and Liliw). The field populations surveyed showed high sensitivity to the diagnostic dose rates of 1 ppm (LC_95_) and 5 ppm (5X LC_95_) (Edralin et al., 2011). A future shift to significant survivorship (i.e. >20%) at the higher rate would indicate incipient problems and a greater risk of resistance developing. Similar baseline monitoring for both chlorantraniliprole and flubendiamide were conducted in Thailand from 2008 to 2010 (Sukonthabhirom et al., 2011), with susceptible field populations from Tub Berg, Petchabun Province, displaying approximately similar LC_50_ values to those reported in the Philippines survey (Table 1).

**Table 1:** Baseline susceptibility of field strains of *P. xylostella* to diamide insecticides.

The baseline susceptibility to chlorantraniliprole in China was established using 16 geographically distinct field populations of *P. xylostella* collected during 2008–2009 from the principal vegetable producing areas, and all field populations were susceptible, with a narrow variation in LC_50_ among populations (Wang et al., 2010). Similar data has also been collected for susceptible field strains from Brazil (Ribeiro et al., 2014, da Silva et al., 2012) and Japan (Steinbach et al., 2015).

## 3 Diamide resistance development

Diamondback moth larvae are historically notorious for the speed at which they can develop resistance to new products. This is probably due to their genetic plasticity, a rapid generation time, high fecundity, and the fact that new chemistry is often heavily used, creating high selection pressure in the field. It has been recorded that *P. xylostella* has developed resistance to 93 insecticides (Whalon et al., 2016) and has become one of the most problematic pests to control in cruciferous vegetables. Flubendiamide (Fenos^®^) and chlorantraniliprole (Prevathon^®^) insecticides were registered in the Philippines in 2006 and 2007, respectively and Voliam Flexi^®^, a premix of chlorantraniliprole and thiamethoxam (a neonicotinoid insecticide) in 2008. Flubendiamide (Takumi^®^ 20WDG) was registered in Thailand in May, 2007. These diamides offered growers excellent control of diamondback moth larvae in cruciferous crops, where few other registered products were adequately effective (Andaloro et al., 2011).

The *P. xylostella* population in Thailand first showed evidence of resistance to flubendiamide (and cross-resistance to chlorantraniliprole) just 18 months after flubendiamide was launched. Field observations in 2009 at Bang Bua Thong, Nonthaburi Province, indicated that Takumi^®^ was not providing adequate control. Resistance factors for flubendiamide and chlorantraniliprole in larvae reared from a field population collected in Sai Noi, Nonthaburi Province (a vegetable growing area near Bangkok), were 66.3 and 35.4 respectively in 2010 (Table 2).

**Table 2:** Resistance development in field strains of *P. xylostella* to diamide insecticides.

In 2011, the Sai Noi field population showed even higher resistance to flubendiamide (RF = 407.2) and chlorantraniliprole (RF = 152.7). Concomitantly, a Tha Muang population, Kanchanaburi Province, showed a very high increase in resistance to flubendiamide (RF = 4817.4) and high resistance to chlorantraniliprole (RF = 87.7), while a field population collected from Lat Lum Kaew, Pathum Thani Province showed an exceptionally high resistance to flubendiamide (RF = 26,602) and high resistance (RF = 775) to chlorantraniliprole. Since the field recommended dose for flubendiamide treatment of *P. xylostella* is only 60 mg/liter, and the Tha Muang and Lat Lum Laew populations had much higher LC_50_’s of 771 mg/l and 4256 mg/l respectively, and the Sai Noi population an LC_50_ of 65 mg/l, this provided a strong indication of resistance being present (Table 2). These field control failures were followed up by laboratory testing, which confirmed the lack of control as being due to resistance development. Some of the key factors identified as leading to diamide resistance in Thailand were an over-dependency on a single mode of action, minimal crop rotation (due to continuous plantings of crucifers), under-dosing with insecticide (to save on cost), irrigation practices that led to excessive product wash-off (providing opportunities for insect exposure to sub-lethal levels), and a lack of any coherent insecticide resistance management (IRM) strategies (Sukonthabhirom et al., 2011). It was found that Thai farmers had used flubendiamide more than 4–5 times per crop in tank mixes with other insecticides for the simultaneous control of *P. xylostella* and other pests in order to reduce the labour costs associated with spraying.

In September of 2009, field representatives covering the Cebu area of the Philippines received reports of reduced control of *P. xylostella* using diamides. Subsequently, throughout 2010, further field failures were reported. Susceptibility monitoring from multiple locations in Cebu Province showed low mortality rates for both chlorantraniliprole and flubendiamide at the highest diagnostic dose rate of 5 ppm and cross resistance of *P. xylostella* larvae to both diamide products appeared evident. Additional monitoring at locations in Negros Oriental also showed reduced susceptibility at 1 and 5 ppm compared to earlier assays conducted from the northern islands (Edralin et al., 2011). However, more than 2 years after being introduced, flubendiamide and chlorantraniliprole were still providing good control against *P. xylostella* in the highlands of Benguet (Edralin et al., 2011). This may have been because the climatic conditions of the two locations differ considerably: the highlands of Benguet have a mean temperature range of 18.5–23 °C, whereas for the midlands of Cebu the main crop production areas have warmer mean temperatures of 25–28 °C. Under warmer temperatures the total life cycle of *P. xylostella* tends to be shorter (Talekar and Shelton, 1993), leading to higher selection pressures. In Cebu province an over-dependency of growers on Fenos® and Prevathon®, continuous planting of related cruciferous crops, the presence of alternate hosts throughout the year, over and under – dosing and a lack of crop rotation were some of the key factors that were identified as having contributed to the development of diamide resistance in the Philippines (Edralin et al., 2011).

In 2011 resistance to diamide products in *P. xylostella* was reported in Taiwan, India (IRAC Newsletter 33), and in the vegetable production area of Guandong Province, Southeast China (Wang and Wu, 2012). During the following years reports of resistance were received from numerous other locations within Asia and resistance was also documented in Brazil and the United States (Mississippi) (IRAC Newsletter 33). It appears that the stability of diamide resistance in *P. xylostella* differs between various field strains. In a highly chlorantraniliprole resistant strain collected from Zengcheng, Guangdog Province, China, an initial high level of resistance (2040 fold, compared to the Roth susceptible strain) quickly dropped to just 25 fold when the selection pressure was withdrawn (Wang et al., 2013a). A rapid decline in resistance (from >27,000 fold to 4000 fold) was also observed in a field collected Brazilian population (Camocim-PE) within just three generations in the absence of selection (Ribeiro et al., 2014). However the Sudlon strain from the Philippines has been shown to maintain a high level of resistance without any further diamide selection (Steinbach et al., 2015).

## 4 Mode of action of diamides

Flubendiamide and chlorantraniliprole act by selective activation of the ryanodine receptor (RyR) in the endoplasmic reticulum of insects. The function of these specialized channels is the rapid release of Ca^2+^ from intracellular stores, which is necessary for muscle contraction. Diamide insecticides induce ryanodine-sensitive cytosolic Ca^2+^ transients independent of the extracellular Ca^2+^ concentration (Nauen, 2006, Ebbinghaus-Kintscher et al., 2006, Ebbinghaus-Kintscher et al., 2007, Cordova et al., 2006, Lahm et al., 2007). This potent activation of RyRs results in a fast initial efficacy in the insect larvae, with an unique symptomology of irreversible muscle contraction paralysis and characteristic feeding cessation (Nauen, 2006).

Radio-ligand binding studies conducted with the 3 commercialised diamides—flubendiamide, chlorantraniliprole and cyantraniliprole revealed species- and order- specific differences in their binding profiles to the RyR in insects. In isolated thorax muscle membranes, from the dipteran *Musca domestica* and the hymenopteran *Apis mellifera*, a high affinity RyR binding site was characterised for the anthranilic diamides chlorantraniliprole/cyantraniliprole but not for the phthalic diamide flubendiamide (Isaacs et al., 2012, Qi et al., 2014). Direct comparison of diamide binding profiles of native muscle membranes from *M. domestica* and those from *Heliothis virescens* and *Agrotis ipsilon* indicated that in Lepidoptera both flubendiamide and chlorantraniliprole compete for the same binding site on the RyR (Qi et al., 2014, Qi and Casida, 2013). Novel diamide actives, constituting sulfoximines and sulfonimidoyl derivatives, show a similar high affinity to insect RyRs as the already marketed compounds (Gnamm et al., 2012). It is also clear from these studies that the binding site for diamides is different and distinct to that for ryanodine on the receptor.

## 5 Biological effects of diamides

The biological effects of sub-lethal doses of chlorantraniliprole on two Brazilian populations of *P. xylostella,* a laboratory susceptible (Recife-PE) and a highly diamide resistant field collected strain (Camocim-PE), were measured following exposure to quantities of the insecticide equivalent to LC_1_, LC_10_ and LC_25_. Insects from both the susceptible and field resistant population had an increased duration of their larval and pupal phases and a reduction in weight, but no significant differences in pupal viability when exposed to the sub-lethal doses. The resistant insects also had significantly lower larval weight and fecundity and higher larval and pupal phases when not exposed to chlorantraniliprole, indicating that fitness effects are associated with the resistant phenotype (Ribeiro et al., 2014). Additionally, females from the resistant population had a significantly higher egg-laying period and longevity at LC_25_, whereas the males lived longer at LC_1_. In an earlier study (Han et al., 2012), exploring the effects of chlorantraniliprole exposure at sub lethal doses (LC_10_ and LC_25)_ on a susceptible laboratory and a susceptible field strain of *P. xylostella* from China, the insects also showed a reduction in pupation rate, decreased pupal weight and delayed adult emergence. The fecundity and survival rates of the emerged insects were also reduced.

## 6 Analysis of *P. xylostella* RyR

Due to its assumed association with rapid resistance development to diamide insecticides, the *P. xylostella* ryanodine receptor (PxRyR) has become one of the best studied (after *D. melanogaster*) insect RyR channels, with a number of groups independently cloning and sequencing the gene encoding the channel (Table 3).

**Table 3:** *P. xylostella* RyR sequences deposited in NCBI.

Accession number	Amino acid length	Reference
AFW97408	5118	Troczka 2012 (Troczka et al., 2012)
AET09964	5164	Wang 2012 (Wang et al., 2012a)
AEI91094	5123	Sun 2012 (Sun et al., 2012)
AFK84956	5131	unpublished
AER25355	5073	Guo 2012 (Guo et al., 2012)
AER25354	5117	
XP_011557207	5123	NCBI Reference Sequences
XP_011562601	4796	

The first complete cDNA of the PxRyR was published in 2012 (Wang et al., 2012a), reporting a gene with an open reading frame (ORF) of 15,495 bp encoding a 5164 amino acid peptide (Wang et al., 2012a). The same study also identified 10 potential alternative splice sites in the sequence, including the well documented mutually exclusive exon pair reported in many other insect species (Wang et al., 2013c, Yuan et al., 2014, Wang et al., 2012b, Liu et al., 2014, Cui et al., 2013). All subsequent published *P. xylostella* RyRs are splice site variants that can be mapped to the original reported sequence (Wang et al., 2012a). A reported deletion between amino acids 870 and 969 (Troczka et al., 2015a), has not been found in other published sequences. Due to the large size of the ORF and the large number of potential splice sites, the exact canonical form(s) of the channel is not known, but it is clear that a 15Kb cDNA encoding a 5118 amino acid protein and incorporating the most frequently reported splice forms, is sufficient to reconstitute a functional channel in insect cell lines (Troczka et al., 2015a). Although a *P. xylostella* genome is available (Tang et al., 2014, Jouraku et al., 2013) it does not have a complete coverage of the PxRyR gene region so there remains a level of uncertainty as to the definitive genomic organization of the receptor. A highly polymorphic gene with significant alternative splicing has not only been reported for *P. xylostella* but also for *Heliothis virescens* (Puente et al., 2000) and other lepidopteran species (Wang et al., 2012a, Wang et al., 2012b, Cui et al., 2013, Wang et al., 2013b). The functional significance of the extensive polymorphisms and alternative splicing on the receptor physiology and whether these are specific specializations in Lepidoptera is unknown.

**Fig. 1:**
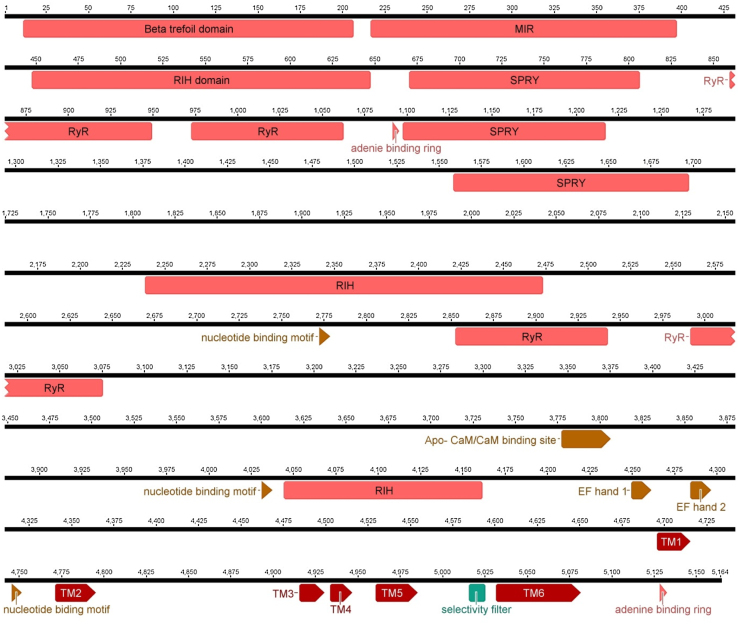
Major protein domains found in *P. xylostella* RyR. The location of these domains is mapped on to the first published PxRyR cDNA sequence (Accession number AET09964) (Wang et al., 2012a). The exact location of each protein domain is presented in Table 4.

At the protein level the PxRyR shares very high amino acid identity (over 90%) with other published lepidopteran RyRs (Wang et al., 2012b, Cui et al., 2013, Wang et al., 2013b, Wu et al., 2013, Sun et al., 2015, Liu et al., 2013, Casper et al., 2010) and a relatively high sequence identity (over 70%) with the RyRs reported from other insect orders. It also incorporates all of the expected features of a ryanodine receptor (Fig. 1, Table 4), including a GVRAGGGIGD selectivity filter located in the channel pore, six predicted transmembrane (TM) helixes (Wang et al., 2012a, Sun et al., 2012), two partially conserved Ca^2+^ binding EF hands motifs and MIR, RyR and SPRY domains (Wang et al., 2012a, Sun et al., 2012, Guo et al., 2012). The TM region of the receptor is highly conserved amongst all insect species (Wang et al., 2012a, Guo et al., 2012) and to a certain extent across the entire animal kingdom (Puente et al., 2000). Seven lepidopteran specific residues, N(4999), N(5001), N(5012), L(5027), L(5058), N(5090), T(5141), located in the TM portion of the channel, are also conserved in PxRyR (Wang et al., 2012b). Recent cryo-electron microscopy imaging of the Rabbit RyR1 (Zalk et al., 2015, Yan et al., 2015) has established the exact relative positioning of the six individual TM helixes within the proteins 3D structure. There is at present no information available for insect RyRs regarding the array of accessory proteins reported to be interacting with mammalian RyRs (Sattelle et al., 2008), and it is unclear whether equivalent proteins exist in insects and if so what is their importance is in the regulation of invertebrate RyRs.

**Table 4:** The location of major protein domains found in *P. xylostella* RyR.

Name	Start	Finish
Beta trefoil domain	12	206
MIR	217	397
RIH domain	448	647
SPRY	671	806
RyR	860	949
RyR	973	1062
adenine binding ring	1092	1095
SPRY	1098	1217
SPRY	1559	1697
RIH	2239	2473
nucleotide binding motif	2773	2778
RyR	2853	2942
RyR	2992	3075
Apo- CaM/CaM binding site	3778	3806
nucleotide binding motif	4032	4037
RIH	4045	4161
EF hand 1	4250	4261
EF hand 2	4285	4296
TM1	4696	4715
nucleotide biding motif	4746	4751
TM2	4772	4795
TM3	4916	4930
TM4	4934	4946
TM5	4961	4985
selectivity filter	5016	5025
TM6	5032	5081
adenine binding ring	5129	5132

## 7 Association of PxRyR variants with resistance to diamides

As reported above, in 2011 two hot spots of diamide resistance were discovered, in Thailand and the Philippines. Strains of *P. xylostella* from these regions were collected from the field and maintained in the laboratory for further analysis. The field-collected strain from Bang Bua Thong, Thailand, was subjected to further selection in the laboratory with chlorantraniliprole. The diamide-resistant (Sudlon) strain from the Philippines was collected in a cabbage field located in Sudlon, Cebu Island. These *P. xylostella* strains showed high resistance ratios to both flubendiamide (resistant ratios of 1300 and 750) and chlorantraniliprole (resistant ratios of 4100 and >200), with the Sudlon strain being the most resistant. Sequencing of the TM region of PxRyR from these strains identified a non-synonymous mutation resulting in a glutamic acid for glycine (G4946E) substitution (Troczka et al., 2012). The precise position of this amino acid change within the TM region of the protein was established by 3D mapping of the TM region of PxRyR to the latest cryo-EM structure of rabbit RyR1 (Steinbach et al., 2015). This showed that amino acid 4946 is located at the junction between TM4 and the TM4-5 linker, on the cytosolic side of membrane, close to the channel pore. Interestingly the coding triplets for this position in the two resistant strains were different (GAG for the Thai strain and GAA for the Sudlon strain), suggesting an independent evolution of the polymorphism rather than a spread associated with migration of the pest between the two countries (Troczka et al., 2012). The G4946E mutation in PxRyR has subsequently been reported as being present in *P. xylostella* in at least 9 countries, spread across 3 continents (Steinbach et al., 2015, Sonoda and Kataoka, 2015).

**Fig. 2:**
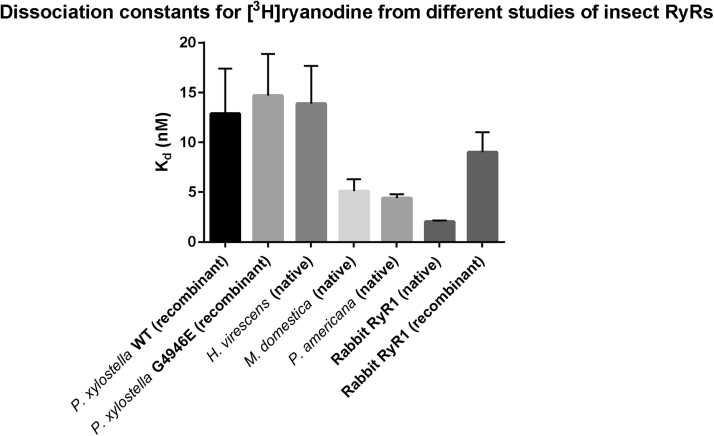
Comparison of mean dissociation constants (K_d_) between the WT and mutant *P. xylostella* RyR (Troczka et al., 2015a) and other insects and rabbit RyR1 reported in literature (Ebbinghaus-Kintscher et al., 2006, Lehmberg and Casida, 1994). The recombinant rabbit RyR1 was expressed in Sf21 cells, the only mammalian channel functionally expressed in insect cell lines (Antaramian et al., 2001).

Follow up research has confirmed the pivotal functional role of G4946E in conferring the resistant phenotype (Steinbach et al., 2015, Troczka et al., 2015a). Functional expression of recombinant PxRyR in Sf9 cells allowed for a comparison of the wild type (WT) receptor and a G4946E modified version. Expressed WT PxRyR was sensitive to caffeine and diamides and was able to bind [^3^H] ryanodine at levels comparable to other insects and mammalian RyR isoforms (Fig. 2).

In contrast the G4946E variant showed greatly reduced sensitivity to diamides, whilst its affinity to other ligands, such as caffeine and ryanodine, remained comparable to WT levels (Troczka et al., 2015a). Similar results were obtained when native microsomal membrane preparations from the susceptible BCS-S (Bayer CropScience reference) and resistant Sudlon strains were used in ligand binding experiments. The affinity of diamide binding to membranes from the Sudlon strain was reduced by over 450-fold for flubendiamide and 159-fold for chlorantraniliprole when compared to the laboratory susceptible strain BCS-S (Steinbach et al., 2015).

The inheritance of G4946E in Sudlon was determined to be autosomal, monogenic and recessive (Steinbach et al., 2015), whereas inheritance of the resistance phenotype in the highly resistant Chinese strain collected from Zengcheng is thought to be autosomal, incompletely recessive and polygenic (Wang et al., 2013a, Liu et al., 2015a). Subsequent sequencing of the Zengcheng strain has confirmed the presence of G4946E (Gong et al., 2014). Additionally the Zhengcheng resistant strain and a second highly resistant strain collected from Guangzhou Bai Yun International Airport, also carrying the G4946E mutation, were both missing an optional splice site (IS10) (Wang et al., 2012a) of 14 amino acids (Q4546 to S4559)(Wang and Wu, 2012, Gong et al., 2014), located 134 amino acids upstream from the first predicted TM helix. This optional splice site is also not present in the most common form of the RyR which was functionally expressed in Sf9 cells (Troczka et al., 2015a).

Additional substitutions, E1338D, Q4594L, I4790M, associated with diamide resistance, have recently been found in the RyR of a *P. xylostella* population collected from Tonghai, Yunnan Province, China (Guo et al., 2014). Frequency analysis of the mutations present in this field population showed that the three substitutions were present in all samples, with 86% of the population being homozygous for the three mutations, whilst the G4946E mutation was only present in a heterozygous form and was only found in 20% of the individuals analysed. The functional implication of these additional mutations with respect to diamide binding and their potential impact on RyR channel kinetics is not at present clear. Mapping of the *P. xylostella* TM region on to the available 3D structure of closed-state rabbit RyR1 shows a very close proximity of G4946E, I4970M and the last 5 amino acids of the 45 amino acid nematode (*Meloidogyne incognita*) RyR cassette which when substituted into a highly divergent region of the Drosophila RyR C-terminus (corresponding to amino acids 4659–4702 in PxRyR) creates a functional, but diamide-insensitive, nematode-drosophila chimeric RyR channel (Tao et al., 2013). The close proximity of these three substituents on the 3D model is suggestive of a possible location for the diamide binding site. However, such a prediction needs to be treated cautiously as the current available RyR models represent the protein in a closed state, to which the diamides do not bind.

So far no metabolic resistance to diamide insecticides has been unambiguously identified in *P. xylostella*. A higher activity of cytochrome P450 enzymes (4.26 times) was reported in a Shan-dong laboratory strain selected with chlorantraniliprole for 50 generations when compared to a non-selected susceptible control, and this could be synergised with piperonyl butoxide (PBO), a known blocker of P450 activity. However no single cytochrome P450 gene was identified as being responsible for the resistant phenotype (Liu et al., 2015b). Small synergistic effects (approximately 2.2–2.9 fold) were also observed in bioassays with PBO and other synergists such as DEM and DEF on a field resistant strain from Zengcheng, Guangdog, China which had been maintained under chlorantraniliprole selection (Wang et al., 2013a). In another study the trancriptome profile of a susceptible strain collected in Guangdong Province, China was compared with a range of chlorantraniliprole resistant field strains collected in Liuzhou, Guangxi Province and Lianzhou and Huizhou, Guangdong Province (characterised as low (5.87 fold), moderately (34.65 fold) and highly resistant (1749.96 fold) respectively). This study identified differentially expressed transcripts associated with insecticide resistance including GSTs (Glutathione-S-Tranferases) and P450s; however there was no clear functional association established between these genes and the resistant phenotype (Lin et al., 2013).

## 8 Regulation of RyR mRNA levels on exposure to diamides

Under normal conditions the expression level of PxRyR changes throughout the life cycle of *P. xylostella*. 2^nd^ instar larvae and adults have the highest PxRyR transcript levels, whilst pupa and prepupa have the lowest (Wang et al., 2012a, Guo et al., 2012). PxRyR mRNA is also differentially expressed in various body parts, with the highest levels being found in the head and thorax of 4th instar larvae (Wang et al., 2012a), At the individual organ level the highest expression of PxRyR mRNA is found in the body wall muscles and the brain (Guo et al., 2012). Differential life stage and tissue specific expression of RyR has also been reported in other insect species (Wang et al., 2013c, Yuan et al., 2014, Cui et al., 2013, Wang et al., 2013b, Yang et al., 2014, Wang et al., 2015). Only in the aphid *Myzus persicae* has no significant transcript level variation between different life stages been identified (Troczka et al., 2015b).

It appears that exposure to diamides may affect transcript levels of the PxRyR gene (Sun et al., 2012, Gong et al., 2014, Liu et al., 2015b, Lin et al., 2013, Yan et al., 2014, Li et al., 2015), transcripts being either up or down-regulated, but whether these observed changes are just a result of diamide-induced toxicity and constitute an insect defence mechanism or whether they may be the basis of resistance development remains unknown. Inconsistent experimental design might be one of the factors contributing to the apparent contradictory results reported in the literature for laboratory susceptible, diamide selected or various field collected diamide resistant *P. xylostella* strains. In some cases the altered transcript levels could be due to modifications to the promoter region, which have been shown to affect gene expression linked with resistant phenotypes (Bass et al., 2013). However, no link has yet been established between transcript levels and the abundance of the PxRyR protein in native membrane preparations, so an apparent increase in transcript level may not necessary facilitate an increase in actual receptors (Maier et al., 2009, Gygi et al., 1999).

## 9 Summary

Diamide resistance is a growing problem globally. This review has focused on the diamondback moth *P. xylostella*, which was the first insect pest to develop resistance to diamides and where the molecular basis of the resistance has been most extensively studied. However, there are still significant gaps in our understanding of resistance in this species. Target site mutations on the RyR are clearly involved in conferring resistance to diamides and there are also indications that there may be a metabolic component contributing to the resistant phenotypes. Subsequent to these studies, control failures relating to diamides have also been reported in other lepidopteran pests. High levels of diamide resistance are present in the tomato leafminer *Tuta absoluta* (Roditakis et al., 2015) collected in Sicily (Resistance factor >2000 fold) and in the smaller tea tortrix *Adoxophyes honmai*, (Resistance factor 77–105 fold) collected in Shizuoka Prefecture, Japan (Uchiyama and Ozawa, 2014). With further active chemicals such tetraniliprole and cyclaniliprole (Sparks and Nauen, 2015), soon to enter the market, selection pressure for resistance development can only increase in the absence of viable and sustainable integrated pest management practices.
